# Harmonising outcome measurement for child focused domestic abuse interventions. Reflections on the development and implementation of a core outcome set

**DOI:** 10.3389/fpsyt.2024.1296437

**Published:** 2024-03-11

**Authors:** Emma Howarth, Gene Feder, Christine Barter, Claire Powell

**Affiliations:** ^1^ School of Psychology, University of Sussex, Brighton, United Kingdom; ^2^ School of Social and Community Medicine, University of Bristol, Bristol, United Kingdom; ^3^ Connect Centre for International Research on Interpersonal Violence and Harm, School of Health, Social Work and Sport, University of Central Lancashire, Preston, United Kingdom; ^4^ Institute of Child Health, University College London (UCL), London, United Kingdom

**Keywords:** domestic abuse, child maltreatment, core outcome sets, interventions, family health

## Abstract

There is appetite in the UK to better measure the impact of domestic violence and abuse (DVA) interventions on children. The spread of outcomes-based commissioning means outcome measurement is no longer just the territory of academic researchers but is now firmly within the purview of practitioners and policy makers. However, outcomes measured in trials only partially represent the views of those delivering and using services with respect to how success should be defined and captured. Even within trials there is huge inconsistency in the definition and measurement of important endpoints. This yields a body of evidence that is difficult to make sense of, defeating the ends for which it was produced – to improve the response to children and families who have experienced abuse. Development of Core Outcome Sets (COS) is seen as a solution to this problem, by establishing consensus across key stakeholder groups regarding a minimum standard for outcome measurement in trials, and increasingly in service delivery contexts. To date COS development has addressed outcomes relating to health conditions or interventions, with limited application to public health challenges. We reflect on our efforts to develop a COS to evaluate psychosocial interventions for children and families experiencing DVA. We highlight the value of COS development as a mechanism for improving evidence quality and the response to families experiencing abuse. Finally, we make recommendations to researchers and COS guideline developers to support this broader application of COS methodology.

## 1 Introduction

Domestic violence and abuse (DVA) is threatening behaviour, violence or abuse between adults aged 16 years and over who are relatives, partners or ex-partners (1). It is a breach of human rights as well as a major public health problem (2). It can occur in any relationship regardless of gender or sexual orientation, although women, transgender and gender non-binary persons are at increased risk of experiencing IPV. It is widely acknowledged that children’s exposure to DVA is widespread and can lead to serious and long-term negative consequences, stretching across all domains of health and development (3–6). This has resulted in government policies to ensure that health and social care services respond to and safeguard children (and their families) who might be at high risk of or have experienced DVA (7–10) However, there is scant high-quality evidence about which interventions are effective and for whom, in which circumstances (11–13).

The current evidence base is limited partly because of the range of outcomes and measures used in DVA evaluations (11, 13, 14). This makes comparing the evidence between and across interventions more difficult. This issue also impacts practice-based research, where funders have been able to draw limited conclusions about the value of multi-million programmes of work (15). Consequently, regardless of the context in which research or evaluation is undertaken, decision makers are unable to draw on evidence to steer decisions about what services to commission. If the point of research is to create real world impact, then this represents a huge waste of resources (16).

More fundamentally, the outcomes measured in intervention studies - particularly trials - do not always reflect concepts of success for those who use, deliver or pay for interventions (17, 18). Typically, outcomes measured in trials reflect the priorities of researchers and are only a partial reflection of what is important to other stakeholders. Since the goal of intervention studies is to understand which interventions benefit individuals, families, and communities, it is crucial that the outcomes measured reflect their priorities. Outcomes also need to be relevant to policymakers and service providers, so that effective interventions are funded and commissioned (17).

One approach to harmonising outcome measurement, whilst bringing together stakeholder priorities on what to measure, is to develop a core outcome set (COS). This is a standardised set of outcomes that researchers, providers, service users, and commissioners agree is important to evaluate the success of an intervention for a health condition or in this case, a complex public health challenge (19). The COS is then measured and reported, as a minimum standard in trials and evaluations and ideally practice-based monitoring too (20, 21). The aim is to improve research practice and reduce wastage, by increasing consistency and reducing reporting bias (where only favourable outcome effects are reported) and ensuring the views of all relevant stakeholders influence outcome selection. While the number of COSs being developed has increased (21), studies have focused on COS development for specific medical conditions, pharmacological, or surgical interventions delivered by healthcare professionals. By contrast, there has been less focus on the development of COSs in relation to public health problems like IPV that typically require complex, multi-agency responses.

Driven by our own experiences of trying to synthesise trial evidence to draw meaningful conclusions about effectiveness (13), as well as an increasing appetite for outcomes measurement amongst UK policy makers, in 2019 we set out to develop to develop two discrete COSs for psychosocial interventions aimed at improving outcomes for children and families at risk or with experience of (1) child maltreatment (CM) or (2) DVA. This saw us attempt to take a health-focused method and extend and adapt it to yield outcomes sets that i) were meaningful to the full breadth of psychosocial interventions on offer to these populations of children and families, as well as the multitude of systems and professionals (beyond health) involved in delivering the response, and ii) privileged the views of people with lived experience of abuse with respect to how the success of interventions should be defined.

In this paper we reflect on key aspects of the project so that others might be able to benefit from our learnings and consider ways of supporting COS development in fields beyond health. We focus specifically on development of the DVA-COS, as recent acknowledgement of children as primary victims of DVA (rather than secondary victims) in the UK has driven a strong policy ‘pull’ for this work, meaning it is more advanced.

## 2 Reflections

### 2.1 A broad scope

We set out to produce an outcome set that could be used to evaluate (in practice or research contexts) any interventions delivered to children or family members, with the aim of improving outcomes for children (<19 years) with experience of, or at risk of experiencing DVA. It is worth restating that a COS is intended as a *minimum standard* and that other outcomes specific to a given programme or population, can be measured alongside.

The scope for our work was necessarily broad to ensure its relevance to the range of interventions on offer which purport to enhance outcomes from children experiencing DVA, as well as the range of stakeholder groups and settings involved in responding to this group (11, 12, 22, 23). On this point we were met with sustained resistance from intervention developers and academic colleagues alike. They argued that different programmes would be characterized by different theories of change, and therefore it would not be possible to ‘prescribe’ a set of outcomes that could be relevant to all interventions. We responded to this argument in several ways. First of all, it presumes available interventions are carefully theorized and described, with clear links drawn between the components of the intervention and intended outcomes. However, DVA interventions are often poorly described with no explicit link to theory, or between activity and outcomes (13, 24). Second, the aims of programmes are often similar, and therefore it is plausible that programmes seek to change similar outcomes, even where mechanisms of change are different (24). Third, the field is already to some extent evaluating effectiveness against some common outcomes – for example, internalising and externalising behaviours– sometimes with no clear theoretical rationale for doing so. Moreover, these outcomes are defined by researchers, privilege measurement of mental health symptoms and diagnoses, and overlook other important aspects of functioning that are important to children and their families (23, 25).

We found it important to emphasise that a COS is intended as a *minimum standard*, with no expectation that an intervention should bring about change in *all* outcomes included in a COS. By articulating the mechanisms through which change in any outcomes are expected to be achieved, it can be made explicit why changes in some outcomes may not be plausible. Understanding which outcomes are *not* changed by a given intervention is just as informative as understanding those which are, in terms of guiding decisions about commissioning and selection. We also challenged developers (and sometimes our academic colleagues) to consider what it would mean in terms of an intervention’s relevance to this population if it would have no plausible effect on *any* of the outcomes included in the final COS. The involvement of multiple stakeholder groups, particularly those with lived experience, and the use of consensus methods to select outcomes, added weight to this line of argument.

### 2.2 Involvement of key stakeholder groups in outcome elicitation and prioritisation

**Figure 1:**
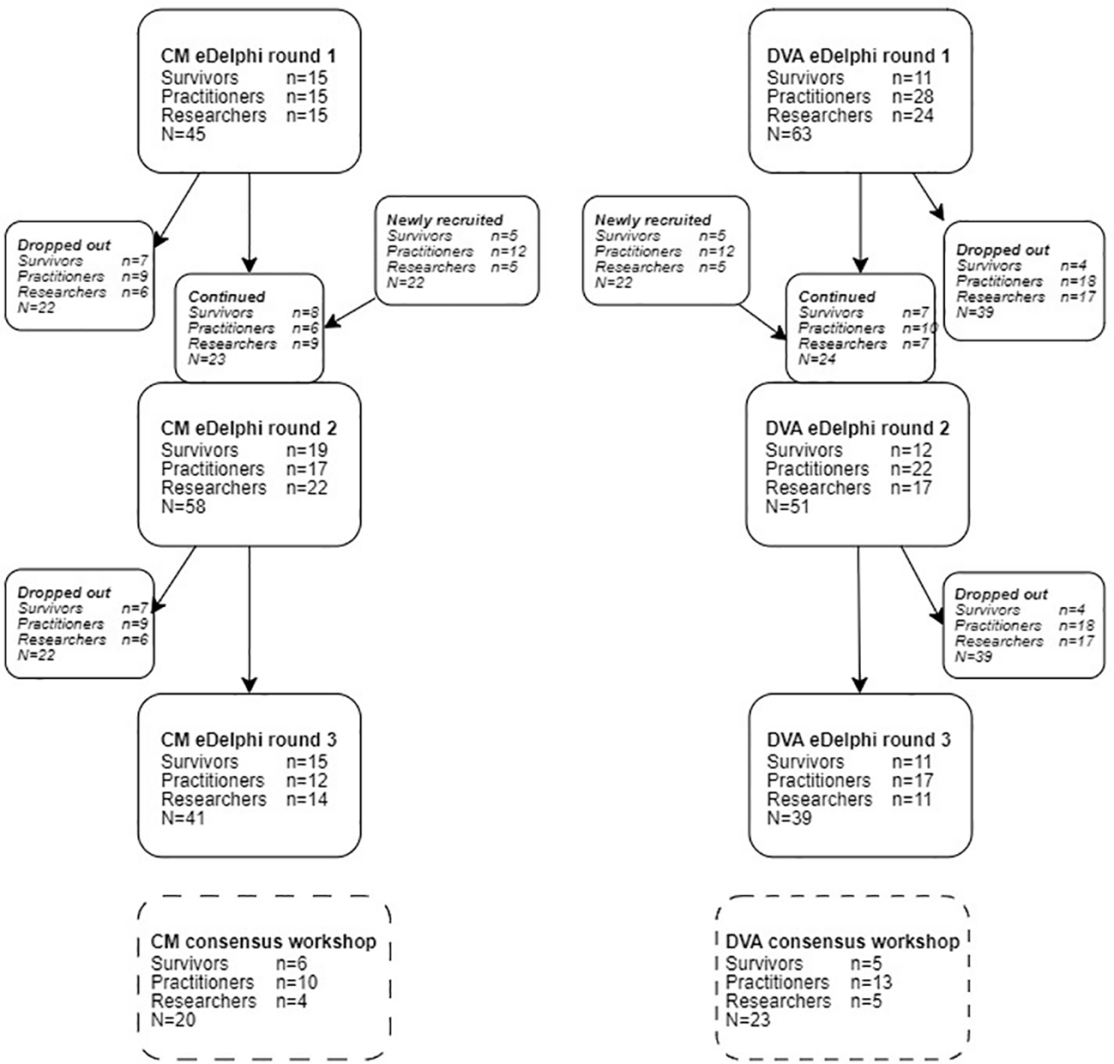
Consensus process participant flow chart. Reproduced from: Powell C, Feder G, Gilbert R, Paulauskaite L, Szilassy E, Woodman J, et al. Child and family-focused interventions for child maltreatment and domestic abuse: development of core outcome sets. BMJ Open [Internet]. 2022;12(9):e064397. Available from: https://bmjopen.bmj.com/content/12/9/e064397 No changes were made and re-use permitted under CC BY. Published by BMJ. https://creativecommons.org/licenses/by/4.0/.

We are applied researchers, and, in this tradition, we sought to involve key stakeholder groups in all stages of the work. We were surprised at the enthusiasm of those with lived, service delivery and strategic experience to contribute to what could have seemed to be a ‘dry’ and methodologically focused endeavour. The DVA sector in the UK is chronically underfunded and we anticipated some ‘push back’ about the use of public funds for this upstream work that could have felt removed from direct service delivery. And whilst there were points of tension, overwhelmingly there was agreement that this was worthwhile and much needed work. We think that the project’s policy relevance and a general focus on outcomes-based commissioning and evidence informed decision making contributed to stakeholder receptiveness. This was reflected in study participation (see **Figure 1** for a summary of stakeholder involvement at each stage of the study) – in our final two consensus workshops a quarter of participants were policy and commissioning stakeholders from a range of central government departments and local authorities. [The remaining 77% was fairly evenly split between survivors, statutory and non-governmental practitioners, and academics. See (18)].

We were less successful in engaging researchers, particularly those outside the UK. We are taking steps to increase awareness of the COS amongst research communities however there is a risk that it is seen as UK specific and less relevant to international colleagues. As most trials are conducted outside of the UK, this may limit its impact on the unification of outcomes measured in effectiveness studies. Having said this, the study was funded by the National Institute of Health and Care Research to support development of the UK evidence base, and so we need to be realistic about our ability to gain traction with international researchers, although others have shown this is possible (21).

In developing our initial protocol, we found there was limited guidance on involving patients, service users or members of the public in COS development or in multistakeholder consensus studies more generally (26). This perhaps reflects the more limited involvement of patients or members of the public in COS development (21, 27). We held an in-person workshop at the beginning of the COS development process, where the aim was to bring together survivors, practitioners and researchers to discuss and define outcomes, generate outcomes as a group and understand how different participants might prioritise outcomes. However, as others have flagged, we encountered some challenges in the bringing together of multiple groups for this purpose (28). We explore these in more detail in a forthcoming publication (26) but we learned that workshops involving trauma survivors and relevant professionals could be distressing without proper planning and support. We were able to use this learning to inform the planning of the final multi-stakeholder consensus meeting held at the end of the process (see below).

Whilst it is becoming more common to involve public representatives in outcome elicitation processes, it is less common to involve them in prioritisation of outcomes (29). To this end we used a modified e-Delphi study design (30). We thought that it could be more difficult to recruit survivors than professionals and this could result in survivor voices being lost in a mixed panel. In anticipation of this, we ran separate e-Delphi studies for each stakeholder panel. This enabled us to track recruitment more closely and recruit additional participants in the second round where needed. To orientate the other panel members to survivor viewpoints, we provided feedback on the survivor panel ratings (for each item) to participants in the researcher and practitioner panels (along with the standard information on group and individuals’ ratings), although not vice versa. Outcomes were identified for discussion at the consensus meeting only if there was agreement across all three groups that an outcome was important (i.e. those rated important by only two groups for example were not taken forwards). For full details of participant flow and outcomes prioritized by each group, see (18).

Concerns about response burden (31) meant that we deviated from our initial protocol, in which we intended for participants to rate the importance of individual outcomes across successive rounds of the consensus process. Instead, in the first round of the Delphi we asked participants to rate the importance of outcome domains [groupings of thematically similar outcomes, see (18)], eliminating all outcomes associated with low-ranking domains. This of course may have resulted in exclusion of some important individual outcomes, but this felt like a necessary measure to ensure our methods were inclusive and realistic with respect to people’s time. We gave the opportunity for feedback throughout the e-Delphi surveys, and we aimed to implement possible changes as quickly as possible throughout the process to widen inclusion. Early feedback from the first survey round suggested the mode of delivery and the language used in survey excluded some survivor participants. To mitigate this, we offered additional support to survivors to complete the survey by phone or email in subsequent rounds. This involved a researcher carrying out the survey over the phone, offering clarifying explanations where needed, and inputting responses into the software for the participant, or sending the survey as a word document attached in an email for participants who struggled to access the software. A researcher then entered the data into the software for the participant.

In reflecting on the study there are important learnings that will inform our future endeavours and may be helpful to others looking to involve people with lived experience of the topic at hand, in their development process. Primarily, researchers should not overlook the potential to cause harm through the research process, particularly when working with vulnerable groups such as those with lived experience of abuse, mental health difficulties and bereavement. The marginalisation or exclusion of individuals or groups in the COS development should not be underestimated as a source of harm. Work is currently underway to explore in more detail harms associated with the development process (32). However COS developers could usefully draw on the extensive mental health research co-production and co-design literatures (33, 34), which highlight the importance of knowledge-based practice and lived experience (35) to improve consensus processes, and acknowledge and mitigate power differences between researchers and service providers (36).

Second, full involvement of survivors in multi-method consensus research, alongside researchers and professionals, requires substantial reflection and planning that extends beyond current guidance on COS development or involvement work more generally. It takes time and money, and this should be factored into research budgets. There is need for specific guidance to support this aspect of the COS development process, including principles as basic as reminding researchers and practitioners how to behave and communicate in multi-stakeholder workshops (28).

Thirdly, it is also worth considering specific measures to ensure that the survivor/patient/service user voice is not lost or diluted through the consensus process. We found the input of a lived experience advisory group to be invaluable from this respect, although again this support needs to be properly resourced from the outset. The approach of running separate Delphi studies and providing feedback on survivor ratings seemed to work well although it significantly increased the resource required and there is only limited evidence that this approach enhances other stakeholder views of service user/patient perspectives (37). We support the call for more empirical research on the best ways to support public involvement in COS development.

Finally, we urge researchers committed to involving patient groups of public representatives to be flexible in their approach – parts of the process set out in the protocol may need to change to facilitate or maintain involvement. This should be encouraged, and deviations transparently reported so that others may benefit from learnings.

### 2.3 Use of a range of evidence sources to identify candidate outcomes

Whilst COS guidance places primacy on conducting a systematic review of trials to identify candidate outcomes, it was necessary for us to draw on a wider range of evidence sources including qualitative and grey literature. Although rigorous, systematic reviews of intervention studies may not include outcomes that survivors see as important. Outcomes in trials are more likely to be in line with research and clinician priorities (17, 38), survivor priorities for outcomes are more likely to be reported in qualitative or grey literature. Thus, a focus solely on trials potentially excludes outcomes of importance to survivors. Our review (39) found that more candidate outcomes were identified in the grey and qualitative literature than the trial literature, and that these outcomes were more nuanced. The inclusion of diverse evidence sources has a direct impact on the final selection of outcomes. In our two COSs, three out of the final eight (unique) outcomes were *only* identified in the grey and qualitative literature. Current guidance needs updating to reflect the importance of evidence sources beyond trials, particularly when the COS may be applicable to marginalised groups whose views may not be well understood or reflected in published research.

### 2.4 The final consensus statement

We used a professional facilitator to help us plan and deliver our final meeting, which was held online during the pandemic, and included representatives from all key stakeholder groups (26). We also paid for the services of a trained counsellor who was available during and following the meeting to respond to any distress experienced by participants. Both were key to the meeting’s success which we gauged not just by the output, but from the feedback we received from participants regarding the respectful and inclusive nature of the debate.

During the meeting we sought to reduce the shortlist of outcomes established by the Delphi study to a list of five. Previous discussions with service delivery stakeholders highlighted feasibility of the COS would be impeded if the set was too large. The final DVA-COS is reported in full elsewhere (18) but included: 1) child emotional health and wellbeing; 2) feelings of safety; 3) freedom to go about daily life; 4) family relationships; 5) caregiver emotional health and wellbeing. It is notable that one of the outcomes (freedom to go about daily life) has not yet been measured in quantitative research, suggesting the process did its job in identifying overlooked outcomes that are important to the users of evidence.

It was also significant the COS included adult and child wellbeing, and that these outcomes were favoured over measurement of mental health (it was possible to include both outcomes in the COS). Research highlights that wellbeing and mental health are separate, although overlapping constructs (40), and that wellbeing outcomes, capturing the extent to which an individual is flourishing, are less often measured in trials relative to mental health outcomes, which are concerned with deficits and distress (17, 41). This finding resonates with early discussions with lived experience experts who expressed a desire for research to capture impact in a more holistic, hopeful, and forwards looking way, rather than by reduction to clinical symptoms and diagnoses, which they saw as overly deficit focused. That said, more work is required to further define outcomes included in the COS (as well as identify measurement tools) to enhance conceptual clarity and reduce potential for misunderstanding between researchers and practitioners (42).

### 2.5 Resource

For the reasons outlined above, the costings and to some extent the time frame were higher and longer than other projects listed on COMET and NIHR websites. Complex COS development needs to be adequately and realistically resourced, particularly when thinking about vulnerable groups or any work that has a broad scope and necessarily involves a range of stakeholder groups.

### 2.6 Implementation

One of the key aims of COS development is reduction in research wastage, however a COS study itself is a waste if nobody uses the output (16). Whilst few (relative to the number of COSs) uptake studies have been undertaken, synthesis of available evidence shows use in trials and systematic reviews to be low (16). Key reasons for this include lack of researcher awareness and understanding about relevant COS, a lack of precision in the definition of outcome domains, a lack of consensus on how to measure outcomes included in the COS, and concerns about a lack of stakeholder (including patient/public) involvement in the development process.

Whilst we were proactive in involving key stakeholder groups from the outset of the study, as noted above, we were less successful in engaging researchers in the process, particularly those from outside the UK. There is a risk here that lack of awareness, or a perceived lack of relevance to our international colleagues may prevent uptake of the COS by trialists and other academic researchers. This may be compounded by the fact that much DVA research, particularly with respect to children, seems to be undertaken outside health, in disciplines such as psychology, social work and social policy. Therefore, the COS, as the product of a health method, may be perceived as less relevant by researchers in other disciplines. We acknowledge we need to do more active work (vs passive dissemination) to increase awareness of the research community. However, this takes time and money that as, yet we are still to secure. Our funding only supported the development of what to measure and did not include funds for the ‘how’, which is significant given this is one of the key barriers to implementation (16, 43).

Demand for the COS amongst service commissioners and providers, facilitated by significant policy developments in the UK, has highlighted the (often cited) tension between policy and practice and research (44). A recent programme of government funding for services for children affected by domestic abuse stipulated that programmes would only be considered eligible for funding if they were able to map how interventions may facilitate change in each of the five outcomes included in the COS and agreed to evaluation of impact against the five outcomes. As researchers, this is the type of impact that we are striving for, and such endorsement of a COS can positively influence uptake (45). However, the desire for immediate policy implementation has been a challenge to our desire to run a properly resourced and rigorous measurement tool selection process aligned with current guidance (44). In order to respond to this ‘pull’ for evidence we have needed to undertake some (very) rapid interim work with a group of service providers to identify measurement tools that are ‘good enough’ to support evaluation of a specific programme of work.

We were able to build on previous work to map the COS against practice-based measurement tools commonly used in practice (46). However, this work could not identify measures for three of the outcomes (safety, family relationships and freedom to go about everyday life) that were acceptable to both service users and providers as well as psychometrically sound. Feedback was that tools were deficit focused, sometimes traumatising to complete, and too narrow in focus. Our subsequent searches, although broader in scope, concurred. We found few measurement tools that had been developed specifically to measure outcomes for this population, and little evidence that general tools had been validated for use with children and families experiencing DVA (22, 47). ‘Freedom to go about everyday life’ was a concept that was not captured by any of the tools we reviewed; it seems highly likely that work will be needed to further define this outcome and develop a relevant measurement instrument.

We have come up with an interim set of tools to measure four outcomes in the COS, but there is a risk that this interim way suite of measures is shared and becomes adopted as the final recommendations on measurement, when we have not yet been able to carry out a thorough selection and consensus process, and there is no suitable measure of one of the outcomes. There are no easy answers to this paradoxical situation – as applied researchers we strive to create the ‘pull’ for evidence, but this may mean offering up incomplete findings that become embedded into practice and difficult to update. This could be mitigated to some extent however, if funders committed to funding both the ‘what’ and the ‘how’ of COS development and were realistic in terms of what this will cost. This requires giving explicit permission to researchers to apply for larger amounts than have been awarded for COS development thus far. In fields such as our own, where the funding for intervention development and service delivery often comes from large charitable organisations, development of better ways to measure outcomes cannot simply be an academic led and funded endeavour. There needs to be a much stronger commitment from these bodies to fund and implement core outcomes work as a mechanism to improve the response to children and families though evidence informed decision making.

## 3 Conclusions and recommendations

Although the origins of COS development are rooted in health research, we found this to be an appropriate method for addressing disparate outcome measurement in relation to child focused DVA interventions, whilst also reflecting the perspectives of survivors of abuse as well as other evidence stakeholder. If we can facilitate uptake, we feel there is genuine potential, albeit some way down the line, to improve the service response to children and families experiencing CM and DVA through better evidence informed decision making about what works.

Given COS development has much to offer other disciplines looking to unify outcome measurement within and between academic and practice-based contexts, we suggest current guidance is updated to reflect this wider application. This could be achieved with additional examples and case studies, and explicit acknowledgement of the utility to disciplines beyond health.

We also suggest inclusion of practical guidance to support the full and meaningful involvement of members of the public, particularly those with lived experience of the topic at hand. Finally, we think that much greater emphasis should be given to the use of diverse evidence sources beyond trials, with recognition that this may be particularly important when working on problems that impact underrepresented and marginalised groups.

We recommend that researchers and funders are realistic about the time and money that is required to undertake a development process that represents the views of all important evidence stakeholders (through involvement and review of evidence). To maximise its value and to make it most meaningful, we recommend that involvement work begins as early as possible and draws on a range of methods across the development process (workshops, written updates, informational videos, briefings).

Finally, we advocate strongly that funders commit to funding both the ‘what’ and the ‘how’ of core outcome development. It is a false economy to fund only the identification of key outcomes without developing consensus on which tools should be used to measure them. Indeed, it may contribute to research wastage.

## Data availability statement

Data are available upon reasonable request. Please contact the corresponding author or unit manager (cpru.data@ucl.ac.uk) with enquiries about the data used in this study.

## Ethics statement

Ethics approval was provided by University College London’s Research Ethics Committee for involving research participants (17893/001 & 002) and we were guided by a steering group of eight professionals. The studies were conducted in accordance with the local legislation and institutional requirements. Written informed consent for participation was not required from the participants or the participants’ legal guardians/next of kin in accordance with the national legislation and institutional requirements.

## Author contributions

EH: Conceptualization, Investigation, Methodology, Supervision, Writing – original draft. GF: Conceptualization, Funding acquisition, Investigation, Methodology, Writing – review & editing. CB: Funding acquisition, Investigation, Writing – review & editing. CP: Data curation, Formal analysis, Investigation, Methodology, Project administration, Software, Supervision, Writing – original draft, Writing – review & editing.

## References

[1] GOV.UK. Domestic Abuse Act 2021 (2021). Available online at: https://www.legislation.gov.uk/ukpga/2021/17/contents/enacted.

[2] Krug EG Mercy JA Dahlberg LL Zwi AB. The world report on violence and health. Lancet. (2002) 360:1083–8. 10.1016/S0140-6736(02)11133-0 12384003

[3] Savopoulos P Bryant C Fogarty A Conway LJ Fitzpatrick KM Condron P. Intimate partner violence and child and adolescent cognitive development: A systematic review. Trauma Violence Abuse. (2023) 24:1882–907. doi: 10.1177/15248380221082081 35666939

[4] Berg KA Evans KE Powers G Moore SE Steigerwald S Bender AE. Exposure to intimate partner violence and children’s physiological functioning: A systematic review of the literature. J Fam Violence. (2022) 37(8):1321–35.

[5] Lee H Russell KN O’Donnell KA Miller EK Bender AE Scaggs AL. The effect of childhood intimate partner violence (IPV) exposure on bullying: A systematic review. J Fam Violence. (2022) 37(8):1283–300.

[6] Bender AE McKinney SJ Schmidt-Sane MM Cage J Holmes MR Berg KA. Childhood exposure to intimate partner violence and effects on social-emotional competence: A systematic review. J Fam Violence. (2022) 37(8):1263–81.

[7] McTavish JR Kimber M Devries K Colombini M MacGregor JCD Wathen N. Children’s and caregivers’ perspectives about mandatory reporting of child maltreatment: A meta-synthesis of qualitative studies. BMJ Open. (2019) 9:e025741. 10.1136/bmjopen-2018-025741 PMC6500368 30948587

[8] Lewis NV Feder GS Howarth E Szilassy E McTavish JR MacMillan HL. Identification and initial response to children’s exposure to intimate partner violence: a qualitative synthesis of the perspectives of children, mothers and professionals. BMJ Open. (2018) 8:e019761. doi: 10.1136/bmjopen-2017-019761 PMC5931305 29705757

[9] Mctavish JR Macgregor JCD Wathen CN Macmillan HL. International Review of Psychiatry Children’s exposure to intimate partner violence: an overview Children’s exposure to intimate partner violence: an overview (2016). Available online at: http://www.tandfonline.com/action/journalInformation?journalCode=iirp20. 10.1080/09540261.2016.1205001 27414209

[10] Domestic Abuse Act 2021, chapter 17. Norwich, UK: England and Wales (2021). Available at: https://www.legislation.gov.uk/ukpga/2021/17/pdfs/ukpga_20210017_en.pdf.

[11] Latzman NE Casanueva C Brinton J Forman-Hoffman VL. The promotion of well-being among children exposed to intimate partner violence: A systematic review of interventions. Campbell Systematic Rev. (2019) 15. doi: 10.1002/cl2.1049 PMC8356495 37131508

[12] Barlow J Schrader A Bowen E. Improving outcomes for children with child protection concerns who have been exposed to domestic abuse. London: Foundations (2023). Available at: https://foundations.org.uk/wp-content/uploads/2023/06/improving-outcomes-for-children-with-child-protection-concerns-who-have-been-exposed-to-domestic-abuse.pdf.

[13] Howarth E Moore THM Welton NJ Lewis N Stanley N MacMillan HL. Improving outcomes for children exposed to domestic violence (IMPROVE): An evidence synthesis. Public Health Res. (2016) 4(10):1–342. 27977089

[14] Macdonald G Livingstone N Hanratty J McCartan C Cotmore R Cary M. The effectiveness, acceptability and cost-effectiveness of psychosocial interventions for maltreated children and adolescents: An evidence synthesis. Health Technol Assess (Rockv). (2016) 20:1–508. 10.3310/hta20690 PMC5056338 27678342

[15] Cordis Bright. Review of Domestic Abuse Outcome Measurement Frameworks. London: Cordis Bright (2016).

[16] Williamson PR Barrington H Blazeby JM Clarke M Gargon E Gorst S. Review finds core outcome set uptake in new studies and systematic reviews needs improvement. J Clin Epidemiol [Internet] (2022) 150:154–64. Available from: https://linkinghub.elsevier.com/retrieve/pii/S0895435622001676 10.1016/j.jclinepi.2022.06.016 35779824

[17] Howarth E Moore THM Shaw ARG Welton NJ Feder GS Hester M. The effectiveness of targeted interventions for children exposed to domestic violence: measuring success in ways that matter to children, parents and professionals. Child Abuse Rev. (2015) 24:297–310. doi: 10.1002/car.2408/asset/car2408.pdf?v=1&t=if5ojdbs&s=f7944da494866240b4eb5858dc099e8ec8896b13

[18] Powell C Feder G Gilbert R Paulauskaite L Szilassy E Woodman J. Child and family-focused interventions for child maltreatment and domestic abuse: development of core outcome sets. BMJ Open. (2022) 12:e064397. 10.1136/bmjopen-2022-064397 PMC9486347 36123087

[19] Williamson PR Altman DG Bagley H Barnes KL Blazeby JM Brookes ST. The COMET handbook: version 1.0. Trials. (2017) 18:280. doi: 10.1186/s13063-017-1978-4 28681707 PMC5499094

[20] Clarke M Williamson PR. Core outcome sets and systematic reviews. Syst Rev. (2016) 5:11. 26792080 10.1186/s13643-016-0188-6 PMC4719739

[21] Gargon E Gorst SL Williamson PR. Choosing important health outcomes for comparative effectiveness research: 5th annual update to a systematic review of core outcome sets for research. PloS One. (2019) 14:e0225980. doi: 10.1371/journal.pone.0225980 31830081 PMC6907830

[22] Steele B Neelakantan L Jochim J Davies LM Boyes M Franchino-Olsen H. Measuring violence against children: A COSMIN systematic review of the psychometric and administrative properties of adult retrospective self-report instruments on child abuse and neglect. Trauma Violence Abuse. (2023) 25(1):183–96. doi: 10.1177/15248380221145912 PMC10666516 36695372

[23] Bunce A Carlisle S Capelas Barbosa E. The concept and measurement of interpersonal violence in specialist services data: inconsistencies, outcomes and the challenges of synthesising evidence. Soc Sci. (2023) 12:366.

[24] Rizo CF Macy RJ Ermentrout DM Johns NB. A review of family interventions for intimate partner violence with a child focus or child component. Aggress Violent Behav. (2011) 16:144–66. doi: 10.1016/j.avb.2011.02.004

[25] Johnson L Stylianou AM. Coordinated community responses to domestic violence: A systematic review of the literature. Trauma Violence Abuse. (2020) 23:506–22. doi: 10.1177/1524838020957984 32954993

[26] Powell C Szilassy E Cowan K Feder G Gilbert R Howarth E. Adapting a consensus process for survivors of domestic abuse and child maltreatment: A brief report about creating safety and avoiding harm. (in preparation). 10.1136/bmjopen-2024-090017 PMC11784130 39843367

[27] Goren K Monsour A Stallwood E Offringa M Butcher NJ. Pediatric core outcome sets had deficiencies and lacked child and family input: A methodological review. J Clin Epidemiol. (2023) 155:13–21. 36528231 10.1016/j.jclinepi.2022.12.009

[28] Young B Bagley H. Including patients in core outcome set development: issues to consider based on three workshops with around 100 international delegates. Res Involv Engagem [Internet]. (2016) 2:1–13. doi: 10.1186/s40900-016-0039-6 29507761 PMC5831887

[29] Jones JE Jones LL Keeley TJH Calvert MJ Mathers J Jones J. A review of patient and carer participation and the use of qualitative research in the development of core outcome sets. PloS One. (2017) 12:e0172937. doi: 10.1371/journal.pone.0172937 28301485 PMC5354261

[30] Howarth E Powell C Woodman J Walker E Chesters H Szilassy E. Protocol for developing core outcome sets for evaluation of psychosocial interventions for children and families with experience or at risk of child maltreatment or domestic abuse. BMJ Open. (2021) 11(8):e044431. 10.1136/bmjopen-2020-044431 PMC8383853 34426460

[31] Gargon E Crew R Burnside G Williamson PR. Higher number of items associated with significantly lower response rates in COS Delphi surveys. J Clin Epidemiol. (2019) 108:110–20. 10.1016/j.jclinepi.2018.12.010 PMC6438267 30557677

[32] Tay J. Adverse events in core outcome set development – an investigation of current approaches and development of a ‘meta-COS’ for cancer surgery . Available online at: https://comet-initiative.org/Studies/Details/1764.

[33] Fitzpatrick SJ Lamb H Stewart E Gulliver A Morse AR Giugni M. Co-ideation and co-design in co-creation research: Reflections from the ‘Co-Creating Safe Spaces’ project. Health Expect. (2023) 26:1738–45. 10.1111/hex.13785 PMC10349236 37254844

[34] Zechmeister-Koss I Aufhammer S Bachler H Bauer A Bechter P Buchheim A. Practices to support co-design processes: A case-study of co-designing a program for children with parents with a mental health problem in the Austrian region of Tyrol. Int J Ment Health Nurs. (2023) 32:223–35. 10.1111/inm.13078 36226745

[35] Glasby J Beresford P. Commentary and Issues : Who knows best? Evidence-based practice and the service user contribution. Critical Social Policy. (2006) 26:268–84. doi: 10.1177/0261018306059775

[36] Douglas N Hinckley J Grandbois K Schliep M Wonkka A Oshita J. How a power differential between clinicians and researchers contributes to the research-to-practice gap. American Journal of Speech-Language Pathology (2023) 32(2):803–10. doi: 10.1044/2022_AJSLP-22-00207 36763851

[37] Macefield R Blencowe N Brookes S Jacobs M Sprangers M Williamson P. Core outcome set development: the effect of Delphi panel composition and feedback on prioritisation of outcomes. Trials. (2013) 14:77.23510124

[38] Keeley T Williamson P Callery P Jones LL Mathers J Jones J. The use of qualitative methods to inform Delphi surveys in core outcome set development. Trials. (2016) 17:230. 27142835 10.1186/s13063-016-1356-7 PMC4855446

[39] Powell C. The importance of grey and qualitative literature in developing domestic violence and abuse and child maltreatment core outcome sets: A brief report. J Family Violence (2023) 31:1–8. 10.1007/s10896-023-00662-z PMC12370783 40862162

[40] Patalay P Fitzsimons E. Correlates of mental illness and wellbeing in children: are they the same? Results from the UK millennium cohort study. J Am Acad Child Adolesc Psychiatry. (2016) 55:771–83. 10.1016/j.jaac.2016.05.019 27566118

[41] Hoagwood KE Jensen PS Acri MC Serene Olin S Eric Lewandowski R Herman RJ. Outcome domains in child mental health research since 1996: have they changed and why does it matter? J Am Acad Child Adolesc Psychiatry. (2012) 51:1241–1260.e2. 23200282 10.1016/j.jaac.2012.09.004 PMC3513697

[42] Verhage ML Tharner A Duschinsky R Bosmans G Fearon RMP. Editorial Perspective: On the need for clarity about attachment terminology. J Child Psychol Psychiatry. (2023) 64:839–43. doi: 10.1111/jcpp.13675 PMC10953320 35916428

[43] Hughes KL Williamson PR Young B. In-depth qualitative interviews identified barriers and facilitators that influenced chief investigators’ use of core outcome sets in randomised controlled trials. J Clin Epidemiol. (2022) 144:111–20. 10.1016/j.jclinepi.2021.12.004 PMC9094758 34896233

[44] Prinsen CAC Vohra S Rose MR Boers M Tugwell P Clarke M. How to select outcome measurement instruments for outcomes included in a “Core Outcome Set” – a practical guideline. Trials. (2016) 17:449. doi: 10.1186/s13063-016-1555-2 27618914 PMC5020549

[45] Hughes KL Kirkham JJ Clarke M Williamson PR. Assessing the impact of a research funder’s recommendation to consider core outcome sets. PloS One. (2019) 14(9):e0222418. 31518375 10.1371/journal.pone.0222418 PMC6743767

[46] Powell C Eyrúnardóttir Clark S Downes L Feder G Fulton E Howarth E. A rapid review of outcome measurement tools related to the DVA Core Outcome Set. London: UCL (2022).

[47] Meinck F Neelakantan L Steele B Jochim J Davies LM Boyes M. Measuring violence against children: A COSMIN systematic review of the psychometric properties of child and adolescent self-report measures. Trauma Violence Abuse. (2023) 24:1832–47. doi: 10.1177/15248380221082152 PMC10240621 35446727

